# Effects of platelet-rich plasma injection for pain control and cartilage repair in knee osteoarthritis: A protocol for the systematic review and meta-analysis of randomized controlled trials in animal models

**DOI:** 10.1097/MD.0000000000024107

**Published:** 2021-01-08

**Authors:** Li Bocun, Li Jing, Li Jia, Qian Tan, Jianyi Chen, Zhongsheng Huang, Cai Guowei

**Affiliations:** a Department of Acupuncture, Union Hospital, Tongji Medical College, Huazhong University of Science and Technology; b College of acupuncture and orthopedics, Hubei University of Chinese Medicine/Hubei Provincial Collaborative Innovation Center of Preventive Treatment by Acupuncture and Moxibustion, Wuhan, China.

**Keywords:** animal models, knee osteoarthritis, meta-analysis, platelet-rich plasma, system review

## Abstract

**Background:**

Knee osteoarthritis (KOA) is a common disabling condition and a heavy financial burden to the society. Platelet-rich plasma (PRP) is considered to be an effective method in the repair and regeneration of cartilage and alleviate pain in KOA. But the utilising of PRP to treat KOA in clinical has shown variable results from many studies. The objective of this protocol is to determine the efficacy of PRP in pain control and cartilage repair in KOA animal models.

**Method:**

We will search the following three electronic databases: MEDLINE, EMBASE and Web of Science. The primary outcome will include the histological score of cartilage and pain score. The secondary outcomes will be the behavioural assessments and cartilage thickness. SYRCLE's risk of bias tool will be used to assessment the risk of bias of including studies. The standardized mean difference and 95% confidence interval will be used to calculate the effect of PRP treatment. The I^2^ inconsistency values will be used to calculated the heterogeneity between studies.

**Results:**

The results of this paper will be submitted to a peer-reviewed journal for publication.

**Conclusion:**

This research will determine the efficacy of PRP of the treatment of knee osteoarthritis model.

**PROSPERO registration number:**

CRD42020181589.

## 1 Introduction

Osteoarthritis (OA) is the most commonly knee joints disease which characterized by degeneration of articular cartilage and subchondral bone accompanied by joint pain, swelling and stiffness, and is a leading cause of disability among older adults.^[1,2]^ The knees, hips, are the most commonly affected appendicular joints and the knee joint appears to be more commonly affected than the hip.^[3]^ Physical approaches, exercise, oral anti-inflammatory drugs are recommended for patients with knee OA in the recent guideline.^[4]^ However, to date it is hard to prevent, slow, or halt OA structural changes, and because of the risk of serious adverse effects from oral anti-inflammatory drugs such as NSAIDs and Opioids,^[5]^ development of more effective therapies that with low risk of adverse effects is expected.

Regenerative medicine is a rapidly growing field and 1 that is fairly new. With the bioengineering technology widespread applied, many researchers are exploring the feasibility of newer technical methods targeting the applications which restoring joint structure and function in OA.^[6]^ As a main approache of regenerative medicine applied in OA, platelet-rich plasma (PRP) is increasingly used for its ability to promote cartilage regulation from the growth factors present in elevated platelet levels, and thereby reduce pain associated with OA.^[7–10]^ PRP which obtained by centrifugation of whole blood, currently, believed to promote chondrogenesis and relieve pain by inhibiting the catabolic cytokines of IL-1β, TNF-α,^[11,12]^ promoting fibroblast growth factor, transforming growth factor-β (TGF-β)^[13–15]^ and alteration of autophagy.^[16]^

Multiple meta-analysis and systematic review have shown that the PRP treatment can be expected to improved clinical outcomes of patients with knee OA, but more randomized controlled trials is needed to determine any regenerative potential of PRP.^[17–21]^ Currently, PRP treatment is still recommended against in patients with knee OA in several guidelines because of the heterogeneity of the evidence as well as lack of standardization in the PRP preparation method and composition.^[4,22]^ Therefore, results of animal studies which promoting the development of treatment approaches for human diseases are used to inform human research.^[23]^ A number of animal experiments have been carried out to study the effect of PRP on the progression of osteoarthritis. systematic reviews and meta-analysis research to date, however, is still scarce. Our study therefore is to summarize the effect of PRP on OA in experimental animals and discuss the translation of this effect to human research.

## 2 Method and design

This protocol is registered in International Prospective Register of Systematic Reviews (CRD42020181589). This is a systematic reviews and meta-analysis of randomized controlled trials in animal studies, so ethical approval is not necessary.

### 2.1 Eligibility criteria

#### 2.1.1 Types of studies

The systematic review will include randomized controlled studies which evaluated the effect of intra-articular injection in the knee of PRP on animal models of osteoarthritis. There is no restriction regarding the language, date, or publication status.

#### 2.1.2 Types of animal models

We will include studies that used any type of animal model, which have developed osteoarthritis genetically, physiological, through drug, surgical, or gene knockout. Each of these models mimics at least part of the various pathophysiological aspects of osteoarthritis.

#### 2.1.3 Types of comparators

Animals whose osteoarthritis was induced but not treated or treated with placebo will be included.

#### 2.1.4 Types of intervention

Animals treated with intra-articular injection in the knee of PRP alone. There is no restriction of dose, concentration and preparing method of PRP. The PRP intervention must be administered during or after the induction of experimental osteoarthritis.

### 2.2 Exclusion criteria

Animals combined with other disease, for example, the ovariectomy model induced to contribute cartilage degeneration. In vitro experiments, case report and all studies in humans will be excluded. Exclusion criteria still comprise studies combined with other intervention like hyaluronic acid or acupuncture, studies using PRP as pre-treatment and studies without a separate control group, pathological and pain score outcomes.

### 2.3 Types of outcome measures

#### 2.3.1 Primary

Histological score of cartilage and pain (mechanical pain and thermal pain) score of hyperalgesia.

#### 2.3.2 Secondary

Behavioural assessments, cartilage thickness.

### 2.4 Search methods for identification of studies

#### 2.4.1 Electronic searches

We will search the following electronic bibliographic databases: MEDLINE, EMBASE, Web of Science. The full search strategy is based on the search components “animal model,” “platelet-rich plasma,” “osteoarthritis.” No publication date, language or publication status exclusions will be applied. The searches will be re-run just before the final analyses to retrieve the most recent studies eligible for inclusion.

#### 2.4.2 Search strategy

The main terms “platelet-rich plasma,” “osteoarthritis,” “cartilage degeneration,” and “Animal Experimentation,” with the Boolean operators “AND or”. Two reviewers independently assessed the titles and abstracts of reports identified by the electronic searches. In case of disagreement between the reviewers, a third reviewer made the final decision. The Research Filter for laboratory animals will also be used.

## 3 Data collection and analysis

### 3.1 Selection of studies

**Figure 1:**
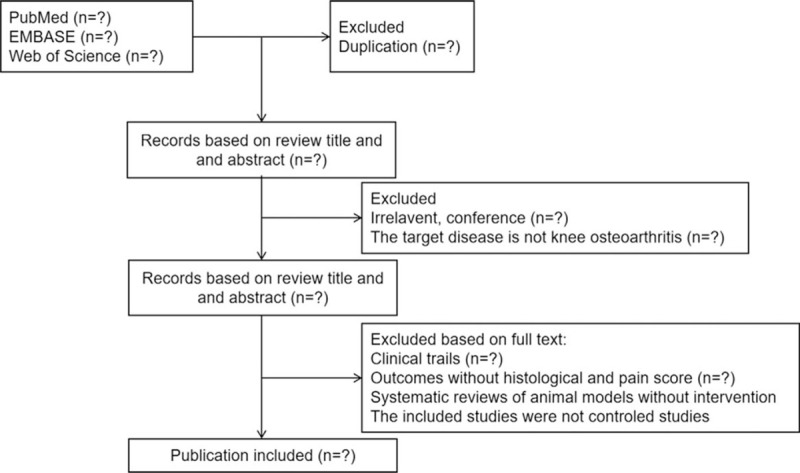
Flow diagram of including studies.

The results of electronic searches will be managed using Endnote X9. Two reviewers (ZSH and QT) will review articles identified from different databases according to eligibility criteria independently. Duplicates will be fist removed. A third reviewer (JYC) made the final decision when there is a disagreement between 2 researchers. A flow diagram will be presented to describe the process of study selection (Fig. 1).

### 3.2 Data extraction

Data will be extracted by 2 independent reviewers and managed by a excel electronic table. The following items will be collected: author, year of publication, study title, sample size and type of sample, animal model, animal sex, age and weight, the dose, concentration, preparation method, time and frequency of PRP injection, Histological score and pain score, In case the data are only represented graphically, the graughs will be translated into data manually using the Image J software version 1.47t (ImageJ, US National Institutes of Health, Bethesda, MD, http://imagej.nih.gov/ij/, 1997–2015). If the data are not reported or unclear, we will attempt to contact the authors by e-mail.

### 3.3 Assessment of risk of bias and quality assessment in included studies

The Systematic Review Centre for Laboratory animal Experimentation SYRCLE's risk of bias tool will be used to assessment of risk of bias and quality assessment in included studies.^[24]^ Selection bias, performance bias, detection bias, attrition bias and other sources of bias will be measured by 2 reviewers (ZSH and QT) independently and any discrepancies will be solved with a third reviewer (JYC).

### 3.4 Measures of treatment effect

Continuous data will be calculated as standardized mean difference, odds ratio (OR), and related 95% confidence interval for each outcome using Cohen method to normalize the different animal species. Dichotomous data will be calculated as risk ratio (RR) with 95% confidence interval.

### 3.5 Assessment of heterogeneity and data synthesis

We will use I^2^ to calculate between-study heterogeneity. Heterogeneity will be definite to following 3 degree, 0% to 25%, 26% to 50% suggest low heterogeneity, 50% to 75% moderate heterogeneity, and >75% high heterogeneity. When the heterogeneity is too high, we will conduct descriptive analysis instead of data synthesis to avoid the adverse impact of excessive heterogeneity.

Considering the exploratory nature of animal study, we will use a random effects model. We will represent the data qualitatively when included studies are not enough, and the meta-analyses will be conducted when there are more than 2 studies. Data synthesis will be carried out using STATA Statistical software version 14.2 (Stata Corp, College Station).

### 3.6 Assessment of publication bias

The funnel plot to evaluate the potential publication bias will be used when there are more than 10 studies included in the meta-analysis. And quantitative analysis of publication bias will measured by the method of Egger test or Begg test.

## 4 Dicussion

Some study have reviewed the effects of PRP in cartilage repair in animal models,^[25–27]^ but to be the best of our knowledge, there is no previous meta-analysis reviewed the efficacy and safety of PRP in animal modeol. To date, there are some problems remain unclear for regenerative medicine in research, and clinical translation. First, the ideal PRP composition and volume such as leucocyte rich versus leucocyte poor PRP, volume of PRP required for knee OA and activated versus non activated PRP is not clearly defined and requires further standardization. Second, there is a conflicting evidence of efficacy of PRP and HA combination from clinical studies.^[28,29]^ Third, PRP is reported to use with biomaterial like gelatin hydrogels, chitosan, polylactic-co-glycolic acid (PLGA) mesh and β-tricalcium phosphate scaffolds and have shown a better effect than PRP alone for knee OA,^[30–32]^ but further study is needed to evaluate its safety and effectiveness. Our study will help to clearly present the most appropriate PRP preparation method and composition in animal model. We hope that the results of this meta-analysis will help clinicians make more appropriate choices in the PRP therapy.

## Author contributions

All authors participated in the review of the manuscript; LBC writes the protocol, JL and CGW conceived and designed the protocol, LBC, JYC, ZSH and QT reviewed the search strategy, JL and QT registered the protocol in International Prospective Register of Systematic Reviews, JL revised the manuscript.

**Data curation:** Li Jing.

**Formal analysis:** Cai Guowei.

**Funding acquisition:** Cai Guowei.

**Methodology:** Zhongsheng Huang.

**Project administration:** Li Jia.

**Resources:** Jianyi Chen.

**Software:** Qian Tan.

**Supervision:** Cai Guowei.

**Validation:** Jianyi Chen.

**Visualization:** Jianyi Chen.

**Writing – original draft:** Li Bocun.

**Writing – review & editing:** Li Bocun.
